# Detecting and monitoring incontinence associated dermatitis: Does impedance spectroscopy have a part to play?

**DOI:** 10.1177/09544119231159178

**Published:** 2023-03-07

**Authors:** Emily J Owen, Rachel A Heylen, Kyle Stewart, Paul G Winyard, A Toby A Jenkins

**Affiliations:** 1 Department of Chemistry, University of Bath, Bath, UK; 2 Watercress Research Ltd., Exeter, UK

**Keywords:** Bioelectric data acquisition, biomedical devices, diagnostic monitoring, dermatology, impedance spectroscopy, incontinence associated dermatitis

## Abstract

In this review, current understanding of the prevention and treatment of Incontinence Associated Dermatitis (IAD) is discussed. The need for preventative measures which target specific faecal/urinary irritants is highlighted, including the role of urease inhibitors. There is no existing internationally and clinically accepted method to diagnose and categorise the severity of IAD. Diagnosis currently relies on visual inspection; non-invasive techniques to assess skin barrier function could remove subjectiveness, particularly in darker skin tones. Impedance spectroscopy is a non-invasive technique which can be used to monitor skin barrier function, supporting visual assessments. Six studies (2003–2021) which used impedance to assess dermatitis were reviewed; inflamed skin was distinguishable from healthy skin in each case. This suggests that impedance spectroscopy could be useful in diagnosis early-stage IAD, potentially enabling earlier intervention. Finally, the authors present their initial findings on the role of urease in skin breakdown in an *in vivo* IAD model, using impedance spectroscopy.

## Graphical abstract

**Graphical abstract:**
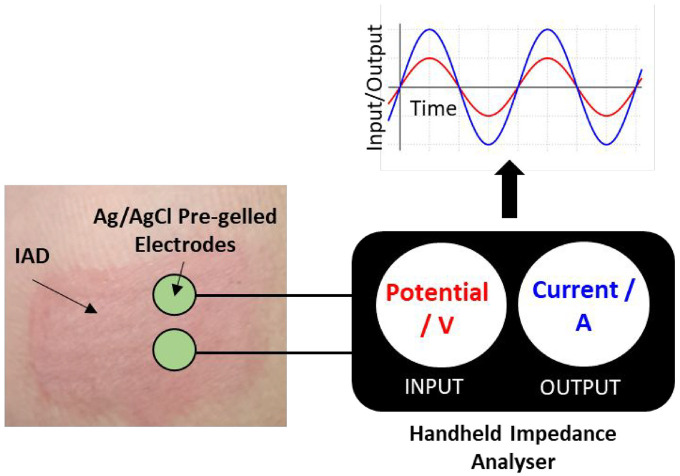

## Introduction

Incontinence Associate Dermatitis (IAD) is a type of skin inflammation resulting from prolonged exposure to urine and/or faeces.^1,2^ The condition is a clinical manifestation of Moisture-Associated Skin Damage (MASD); individuals who experience urinary, faecal or double urinary and faecal incontinence are at risk of developing IAD.^2–5^ Current understanding of the aetiology, pathophysiology and mechanisms of progression of IAD needs to be improved in order to prevent poor patient outcomes.^1,3^ Supporting visual assessment with non-invasive techniques to monitor skin barrier function may improve understanding of the key pathways involved in the manifestation of IAD, allowing earlier diagnosis and intervention.^3,6,7^ Impedance spectroscopy is a non-invasive technique which can be used to characterise biological tissue, including assessment of skin barrier function.^8–10^

The contents of the current review includes the following sections:

1. IAD: Background
a. Clinical Issue
b. Aetiology, Pathophysiology and Mechanisms of Progression
c. Microbiome
2. Management of IAD
a. Current Prevention and Intervention Strategies
b. Future Prevention and Intervention Strategies
3. Detecting and Monitoring IAD: The Potential Use of Impedance Spectroscopy
a. Importance of Improving Detection and Monitoring of IAD
b. Standard Measures of Skin Barrier Function
c. Impedance Spectroscopy: Theory and Application in Measuring Skin Barrier Function
d. Using Impedance Spectroscopy to Monitor non-IAD Dermatitis
e. Using Impedance Spectroscopy to Monitor IAD
f. Limitations of Impedance Spectroscopy
4. Detecting and Monitoring IAD with Impedance Spectroscopy: Exploratory Work and Preliminary Findings
a. Methods and Materials
b. Results and Discussion

### IAD: Background

#### Clinical issue

The formal classification of IAD, according to the WHO International Classification of Diseases 11th Revision (ICD-11), falls under irritant contact dermatitis.^11,12^ Depending on the extent of skin contact with urine/faeces, IAD can spread from the perineum/genitalia/glutaeal fold, reaching as far as the abdomen and thighs.^3^ Whilst IAD poses a significant clinical issue, the extent is unclear. This is perhaps due to a lack of internationally accepted methods to diagnose IAD, reflected by the unsurprisingly vague estimated prevalence of 5.6%–50% and incidence of 3.4%–25% reported in IAD data collection studies.^2,3,6^ An internationally recognised categorisation tool would be very useful.

Patients with IAD commonly experience pain, pruritus (itchiness) and sensations of burning in the affected skin sites. Furthermore, IAD can reduce an individual’s quality of life and affect independence.^3,13^ Whilst IAD is usually not directly life-threatening, it poses risks of developing secondary infections, especially dangerous to frail, immunocompromised individuals or those with complex co-morbidities.^14,15^ Furthermore, it has been suggested that IAD is an independent risk factor for pressure ulcer development in susceptible adults.^14,15^ The presence of erythema or even partial thickness skin loss can be misidentified as Grade I or Grade II pressure ulcers, respectively.^2^ As a result, the clinical presence of IAD can be confused with pressure ulcers; leading to misdiagnosis and mismanagement.^16^ Therefore, by improving IAD prevention strategies, the risk of pressure ulcer formation may also be reduced.^14^

IAD is most commonly reported in very young and elderly patients due to being the primary incontinent populations and having distinct physiological features compared to healthy adult skin, including thinness and fragility.^4,6^ In children, the terms ‘diaper dermatitis’ and ‘nappy rash’ are often used the define the same aetiology as IAD.^4,17^ The relationship between both infant and elderly IAD and neglect/abuse is frequently commented on in the literature,^18^ but there is an absence of published research into this specific correlation, despite severe IAD often being mentioned in child abuse reports.^19^ In terms of the geriatric population, aged skin has a reduced rate of cell replacement, resulting in an approximate 20% loss in dermal thickness and delayed wound healing. Furthermore, the decreased production of sebum, sweat, natural moisturising factors and lipids leads to a compromised skin barrier function.^2,4,6^ With up to 50% of nursing home residents affected by urinary incontinence and 23%–66% affected by faecal incontinence, the risk of developing IAD is significant.^20^ On the opposite end of the scale, infant skin is composed of smaller keratinocytes, a thinner epidermis and faster cell proliferation compared to adult skin.^4,6,7^ Childhood IAD is a relatively common cause of hospital outpatient attendance: in the United States an estimated 4.8 million outpatient visits occurred from 1990 to 1997 (approximately 600,000 per year) for uncomplicated diaper dermatitis and almost double that if related conditions, such as vulvovaginitis and candidiasis, are included.^21^ Although most cases of childhood IAD do not present as hospital outpatients, it is regarded as the most common dermatological disorder of infancy: virtually every child (who has worn nappies for any period) will experience at least on episode of IAD in early childhood.^5,21–24^

#### Aetiology, pathophysiology and mechanisms of progression

**Figure 1.**
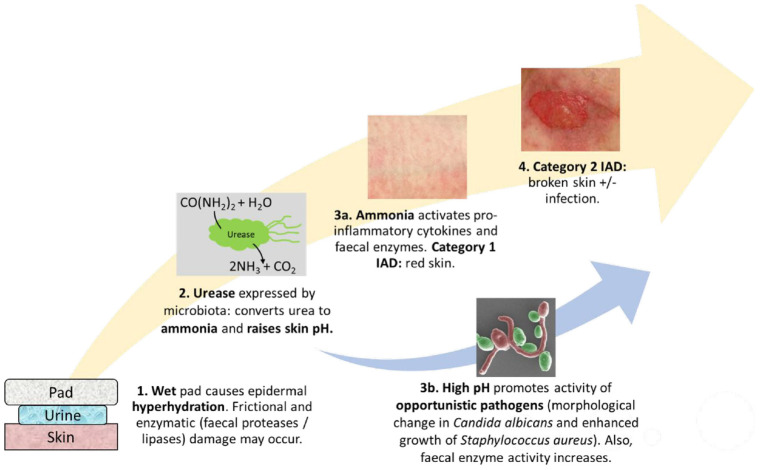
Schematic showing the contributing factors in IAD: wet skin is susceptible to hyperhydration and frictional damage as well as exposure of faecal proteases/lipases, commensal bacterial which express urease and convert urea to ammonia, which chemically damages the skin as well as raising pH. High pH promotes further bacterial growth and increases protease activity as well as causing morphological change in commensal *Candida albicans*, promoting skin degradation.

The aetiology and pathophysiology of IAD is believed to involve several key factors (Figure 1). First, the moist environment created by prolonged exposure of the skin to urine and/or faeces can cause skin maceration. This involves hyperhydration of the keratinocytes and disruption of the intercellular lipid bilayers within the epidermis.^2,3,13^ Prolonged contact with moisture also increases the coefficient of friction (COF), meaning the risk of mechanical damage from the friction generated between the skin and external surface is greater.^3,6,25^ Second, lipolytic and proteolytic enzymes present in the faeces can further damage the skin barrier.^2,13^ These digestive enzymes include trypsin, α-chymotrypsin and lipase which are derived from the gastro-intestinal tract.^13,26,27^ Mugita et al. exposed murine skin to digestive enzymes (trypsin and α-chymotrypsin) and intestinal bacterial species (*Pseudomonas aeruginosa*), observing that proteolytically macerated skin is prone to deeper skin damage by intestinal bacteria compared to other types of contact dermatitis.^13^ This is likely to be because proteases accelerate the transdermal penetration of macromolecules.^28^ Mugita et al. also found that lipidolytic enzymes (lipase and phospholipase A2) may accelerate the transdermal penetration of proteases.^29^

The acidic skin surface of the stratum corneum (pH 4–6) and accompanying steep pH gradient towards the viable epidermis (pH 7.4) is known as the ‘acid mantle’.^6,30^ Urease is a major bacterial virulence factor which catalyses the conversion of urea to ammonia, increasing the skin pH.^7,31^ The elevation in surface skin pH disrupts the natural acid mantle of the skin and causes the stratum corneum to swell.^2^ As a result, the resident flora are no longer in the optimum conditions to thrive and their ability to protect the skin from colonisation of pathogens is diminished, leading to an unfavourable dysbiosis.^32^ Furthermore, the increase in alkalinity increases the activity of faecal enzymes, such as trypsin, leading to accelerated breakdown of proteins and fats in the epidermis.^6,7,31,33^ Chronic exposure to urinary/faecal irritants causes epidermal keratinocytes to release growth factors and pro-inflammatory cytokines, including IL-1α, IL-8 and TNF-α.^6,34,35^ The release of inflammatory cytokines was investigated by Koudounas et al.; 10 healthy participants were exposed to synthetic urine and synthetic faeces and the inflammatory cytokines were collected using the Sebutape absorption method and dermal microdialysis.^35^ If the resulting inflammatory response is prolonged, this can lead to IAD. However, more research is required to understand the relative contributions of each step in the proposed pathway (Figure 1).^16^

There is no internationally and clinically accepted method used throughout clinics to diagnose and categorise the severity of IAD.^2,3,6,36^ The Global IAD Expert Panel (2015) proposed that superficial IAD (Category 1) presents as erythema and/or oedema.^3^ It is important to consider that skin tone influences the visible presentation of early-stage IAD; light skin displays erythema (redness) whilst dark skin may appear darker, paler, purple, red or yellow.^3^ Bliss et al. used an array of photographs and training material to improve distinction of normal and IAD-damaged skin on an array of skin tones.^37,38^ However, supporting visual assessment with non-invasive techniques which monitor skin barrier function would be more effective, particularly in darker skin tones. In more severe cases of IAD (Category 2), papules, vesicles, bullae, maceration, erosion, denudation of skin, ulceration and infection may be present.^3,6,7^ Following on from the proceedings of the Global IAD Expert Panel, in 2018 the Ghent Global IAD Categorization Tool (GLOBIAD) was formally developed by 34 experts as an approach to monitor IAD. GLOBIAD is categorised by persistent redness (Category 1) and skin loss (Category 2), each of which are subcategorised into IAD with/out clinical signs of infection.^39^ After validation of GLOABIAD, the GLOBIAD Monitoring Tool (GLODIAD-M) was developed to monitor the change in IAD status over time. Whilst the GLOBIAD-M has potential to guide treatment of IAD, further validation by clinicians is still needed.^40^ More research is required on the aetiology, pathophysiology and progression of IAD in order to better identify the risk factors and prevent complications arising from severe (Category 2) cases.^1,3^

#### Microbiome

The relationship between skin and commensal flora is a mutualistic symbiosis; skin provides a suitable biotope, while bacteria strengthen the host’s defences against extracellular pathogens.^32^ The low pH inhibits transient bacteria from colonising the skin. However, exposure of the skin to urine and/or faeces alters the ordinary skin microbiome. In particular, faeces contain a range of gastrointestinal bacteria with over 10^11^ bacterial cells per gram.^13^ Bacteria which express urease and proteases are commonly isolated in samples from urinary and/or faecal incontinence patients for example, species of *Proteus*.^26,31,41^ Urease activity in faecal/urinary pathogens can cause a rise in skin pH, allowing opportunistic pathogens to colonise and infect the skin.^32^

*Candida albicans* (*C. albicans*), from the gastrointestinal tract, and *Staphylococcus aureus* (*S. aureus*), from the perineal skin, are the most frequently isolated species in IAD infections.^2,7,20,42^*C. albicans* is a polymorphic fungus; at low pH (<6) cells grow in ovoid-shaped budding yeast whilst at high pH (>7) hyphal growth occurs. The hyphae, which penetrate host cells, are more invasive than the yeast form.^43^*Candida* infections can present as bright red maculopapular rashes or non-specific confluent papules.^42^*S. aureus* infections are characterised by erythematous areas, often with a golden crust and comedones, and occasionally neutrophilic abscesses. The resulting change in the skin microbiome can delay subsequent healing of IAD.^34,44^

### Management of IAD

#### Current prevention and intervention strategies

There are two key IAD intervention strategies, recommended by the Global Expert IAD Panel.^3^ First, incontinence needs to be managed to minimise the exposure of skin to urine/faeces, including frequent changes of incontinence products. In instances where behavioural changes are not enough, this may involve using devices such as urinary catheters or faecal management systems.^3,45^ However, long-term urinary catheterisation carries a high risk of developing catheter-associated urinary tract infections, which can cause extended morbidity and so should only be implemented as a last resort.^46^ Second, a two-step skin care regime should be followed: cleansing and protecting. In terms of cleansing the skin, there is evidence to suggest that the high pH of standard soap can damage skin barrier function.^3,6,45^ This is due to the presence of anionic surfactants, such as sodium lauryl sulphate. Alternative cleansers which contain non-ionic or amphoteric surfactants are believed to be less disruptive to the skin.^3,45^ After cleansing, the skin should be protected from external irritants. Types of protectants include creams, ointments, pastes, lotions and films.^3,20,45^ These formulations are likely to contain some of the following principal ingredients: petrolatum, zinc oxide, dimethicone and acrylate terpolymer.^3,20^ Skin barrier products provide a semi-permeable barrier which help to prevent further loss of water and entry of irritants.^2^

#### Future prevention and intervention strategies

Overall, the recommended intervention strategies involve removing urine and/or faeces from the affected site and applying a protectant. However, they do not target specific irritants present in urine and/or faeces. Until the pathophysiology of IAD, including the role of faecal enzymes, is better understood, targetted therapeutic care cannot be implemented.^29^ Mugita et al. have demonstrated the potential utility of urease inhibitors in preventing the conversion of urea to ammonia and the subsequent rise in skin pH on murine models.^31^ In theory, this could prevent the manifestation of IAD and complications from opportunistic infections, including *C. albicans* and *S. aureus*.^2,7,20,42^ Further novel strategies to prevent IAD should be sought out.

### Detecting and monitoring IAD: The potential use of impedance spectroscopy

#### Importance of improving detection and monitoring of IAD

Currently, the diagnosis of IAD relies on carer/clinical observation, which can be subjective for two main reasons. First, there is no internationally and clinically accepted categorisation tool.^2,3,6^ Second, the presence of erythema can be misidentified as an early-stage pressure injury, leading to misdiagnosis and mismanagement of IAD.^2,16^ There is a need for non-invasive techniques which can be used to monitor skin barrier function and support standard clinical assessments of the skin.^3^ This may allow for earlier intervention and distinction of IAD from visually similar disorders for example, other forms of dermatitis, MASDs, and early-stage pressure injuries.^2,45^ As a result, the appropriate care could be implemented faster. Ideally, there should be development of a device which can provide continuous real-time monitoring of skin health of incontinent individuals who are at risk of developing IAD, via non-invasive techniques. The information could then be transmitted in an easily interpretable form to the clinician/caregiver.^47,48^

#### Standard measures of skin barrier function

Currently, the most conventional non-invasive technique to measure skin barrier integrity is transepidermal water loss (TEWL).^49,50^ An increase in TEWL corresponds to an increase in water diffusion through the skin which indicates skin barrier disruption.^3^ However, differing external environments can cause significant variability in measurements.^49,50^ Furthermore, it has been reported that the interpretation of TEWL measurements outside of a research setting is challenging.^3^ There is a need for further investigations of devices which can be used for the diagnosis of IAD. Investigating TEWL, in combination with other skin parameters, could improve IAD diagnosis and support standard clinical assessments.^3^ Other non-invasive methods to measure skin barrier function include skin surface pH, stratum corneum hydration, colourimetry and sebometry.^50^ Implementing tools which monitor skin barrier function could serve a vital function in IAD research, helping to identify more risk factors that extend the current understanding of IAD pathology. Impedance Spectroscopy could provide an effective way to monitor susceptibility on an individual basis and prevent the progression of IAD.

#### Impedance spectroscopy: Theory and application in measuring skin barrier function

Electrical impedance is the measure of a material’s opposition to the flow of an alternating electrical current.^8^ The technique can be used to non-invasively characterise tissue, including assessment of skin barrier function.^9,10^ Electrical impedance is a generalised form of resistance which take into account the magnitude of the amplitude of an applied voltage (Δ*E*) to a system, divided by the resultant current (Δ*i*) and any difference in phase (*φ*) which results.^8,51,52^ Impedance spectroscopy measures this ratio as a function of the frequency of the applied voltage, often over three or four decades that is, 104–1 Hz. Hence impedance has both a magnitude and a phase, *φ* (equation (1)):

(1) $${\left|Z\right|}_{\omega }=\frac{\Delta E}{\Delta i}\;\mathrm{and}\;a\;\mathrm{phase}\Phi$$

ω is the angular frequency, *2πf*

The impedance of a pure resistor is independent of applied frequency, hence *φ* = 0°; whilst *φ* = 90° for a pure capacitor.^8,52,53^ The measurement of the impedance spectrum of materials, including skin, generally requires the fitting of the spectrum to an equivalent electrical circuit model. This is a simplified model the material, in terms of its resistance(s), capacitance(s) and occasionally inductance(s).^54,55^ In reality, the capacative component of the impedance of many complex materials is a distribution of individual capacitances, which can make fitting the spectrum difficult (equation (2)). In this case, a Constant Phase Element (CPE) can be used, but should be used with care, as CPEs don’t generally have a physical meaning (equation (3)). CPEs often allow a more accurate fitting of the resistance in an R-CPE parallel circuit however, so have some utility.^56^

(2) $$Z_{c}=\;{\left(\omega C\right)}^{-1}$$

(3) $$Z_{\mathrm{CPE}}={\left(\omega C\right)}^{-n}$$

n is the CPE exponent

The two electrical properties which are commonly analysed in skin impedance data are resistance (*R*) and capacitance (*C*).^47^ Cell membranes are semi-permeable, facilitating the passage of selective ions. This behaviour can be likened to an electrochemical membrane, demonstrating capacitive properties. Furthermore, electrolytes present in the intracellular and extracellular spaces result in resistive characteristics.^57^

**Figure 2.**
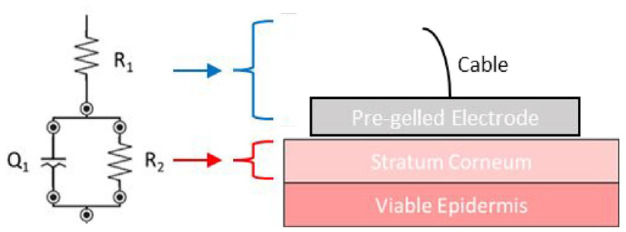
Equivalent circuit of the stratum corneum of skin. R1 pertains to the electrode and cable resistance – and is generally very low; R2 is the stratum corneum resistance and the CPE (denoted as Q) is described in equation (3).

The CPE exponent, *n*, is a parameter which is believed to indicate the degree of electrical heterogeneity of the material under test; n is inversely correlated with this heterogeneity.^58^ When n has a value of 1, CPE is a pure capacitance; when n has a value of 0, CPE is purely resistive. In the context of human skin impedance, n is often considered to have a value of 0.8 and closely relates to skin properties, including water content.^47^ Equivalent electrical circuit models used to represent skin include both RC-based models and CPE-based (RQ) models (Figure 2).^47,59^ R is arguably the most unambiguous parameter to obtain from skin impedance measurements, and when measured by fitting data from the relatively low frequency spectrum (50 KHz–0.1 Hz), primarily relates to the stratum corneum integrity.^47,60^ Hence following the change in measured stratum corneum resistance (*R*_2_) because of, for example, proteolytic activity, pH, ammonia concentration or bacterial inoculum, provides a sensitive method of quantifying the relative effect of these factors on stratum corneum integrity.

Biopotential electrodes are capable of transducing bioelectrical activity (ionic current) present within the body into a quantifiable electrical current.^47,61^ Common non-invasive biopotential electrodes include: silver/silver chloride (Ag/AgCl), Orbital (combination of Ag/AgCl, aluminium, gold/gold chloride, nickel and titanium) and stainless steel.^61^ Ag/AgCl electrodes are universal in clinical settings.^47,48,61–63^

#### Using impedance spectroscopy to monitor non-IAD dermatitis

A scoping review was conducted on 19th September 2022 to determine whether there is any evidence to suggest the utility of impedance as a technique to detect and monitor IAD. Using the advanced search function on Web of Science, 0 results were reported for ‘impedance’ AND ‘incontinence associated dermatitis’. Whilst presently there is no reported study which uses impedance spectroscopy to assess the impact of IAD on skin, there are several examples which monitor dermatitis. Next, the search terms were broadened to ‘impedance’ AND ‘dermatitis’ (2003–2022) which yielded 47 results. Of these results, six original research papers were found to be relevant, in terms of using skin impedance to monitor dermatitis. The studies demonstrate clear potential in using impedance spectroscopy to assess skin barrier function, with regards to dermatitis.

### Measuring impedance indices at frequencies of 20 and 500 kHz

In five of the six studies, the electrical impedance was measured using a skin impedance spectrometer developed by SciBase AB (Huddinge, Sweden), designed by Dr Stig Ollmar.^9,64–67^ The magnitude and phase of impedance at 31 logarithmically distributed frequencies, between 1 kHz and 1 MHz, in a defined volume below the probe (depth settings 1–5, where 1 is the most superficial). The impedance spectrum was analysed in terms of four indices based on data condensed to two frequencies, 20 and 500 kHz. The four indices are defined as:

Magnitude index, $\mathrm{MIX}=\left({\left|Z\right|}_{20\mathrm{kHz}}\right)/\left({\left|Z\right|}_{500\mathrm{kHz}}\right)$

Phase index, $\mathrm{PIX}=\theta \left(Z_{20\mathrm{kHz}}\right)-\theta \left(Z_{500\mathrm{kHz}}\right)$

Real part index, RIX=Re(Z20kHz)/(|Z|500kHz)

Imaginary part index, IMIX=Im(Z20kHz)/(|Z|500kHz)

Where θ is phase angle in degrees, and Re and Im are the real and imaginary components of impedance, respectively, at the specified frequencies.^9,64–67^ Impedance indices are believed to reflect the properties of skin but mechanistic details are not understood at present.^9^

Kuzmina et al. studied the irritation phenomena and recovery over 14 days in 40 healthy participants when exposed to detergents (known skin irritants), 2% SLS and 40% nonanoic acid (NAA), for a 23-h period. Skin impedance was assessed at depth 5 and supported by standard measurements; TEWL, cutaneous blood flow via LDF (Laser Doppler flowmetry) and visual scoring. Inter-laboratory variability was assessed by splitting the participants into two separate laboratories. Both irritants were found to cause distinct responses, in terms of changes in the impedance indices. Both SLS and NAA caused a reduction in MIX but PIX increased with SLS and decreased with NAA. The absolute values varied between the two laboratories but the patterns in impedance data were consistent. Also, an increase in TEWL and LDF was reported upon exposure to both irritants and subsequent recovery, indicating impaired skin barrier function. This work suggests that irritant contact dermatitis reactions can be distinct according to the irritant.^66^

Nicander et al. measured the response of 28 healthy participants to skin damage induced by detergents, due to their tendency to cause contact dermatitis. Soaps with or without the presence of 1% SLS (anionic surfactant) and 1%–10% betaine (amphoteric surfactant) were used. Impedance at all five depths, TEWL and visual inspection were used to assess the skin. In terms of impedance patterns, the exposure to soap was characterised by a decrease in MIX and IMIX, as well as an increase in PIX and RIX at depth 1. No significant changes were reported in RIX at depth 5, perhaps suggesting that more information can be deduced in the superficial skin layers. Furthermore, TEWL increased. Both measurement techniques showed that the presence of betaine caused less skin irritation than SLS. Skin impedance and TEWL were found to be more sensitive than visual inspection.^67^

Kuzmina et al. conducted a study on 29 eczema patients and 19 healthy participants, measuring their response to detergent exposure. Impedance, measured at five depths, showed a clear difference in the baseline properties of healthy and eczema skin types, whilst no significant change was noted with TEWL. Participants were then exposed to 2% SLS for 24 h. It was found that the MIX impedance index decreased and TEWL increased to a greater extent in eczema sufferers compared to the controls. Furthermore, the impedance and TEWL values of eczema patients did not return to baseline at the end of the 7-day measurement period. This could reflect a slower healing rate in eczema patients. In future, it may be possible to monitor the risk of developing irritant contact dermatitis, using impedance as a screening tool.^64^ This study could have been improved by using more than one test irritant, rather than solely SLS. Also, a deeper discussion of the four impedance indices is needed. There was also no mention of distinct differences in measurement depths of impedance.

A study by Nicander and Ollmar involved 22 healthy participants and 26 atopic dermatitis (AD) patients. Impedance, electrical capacitance (stratum corneum moisture) and TEWL were measured. The skin treatment consisted of cyclohexane swabbing, tape-stripping and lipid extraction; measurements were taken in-between each step. Before any treatment, baseline impedance measurements varied significantly between AD and normal skin, contrary to TEWL and stratum corneum moisture measurements. During treatment, tape stripping caused significant decreases in RIX, MIX and IMIX and an increase in PIX on atopic skin at depths 1 and 5 whilst only PIX and RIX displayed significant changes in normal skin. Overall, impedance was more sensitive to changes in AD skin compared to normal skin after tape stripping and lipid extraction. The difference in TEWL values for the two skin types was only significant after tape stripping whilst stratum corneum moisture values were indistinguishable. Overall, impedance displays the clearest distinction in the reactivities of normal and atopic skin of the employed measurement techniques.^65^

A study by Hagströmer et al. involved evaluating the skin of 24 patients with AD, compared to 22 healthy participants. Impedance at depth 2, electrical capacitance (stratum corneum moisture) and TEWL were measured. Prior to any treatment, it was found that certain patterns of electrical impedance were abnormal in the AD patients compared to normal skin.^9^ The IMIX and MIX impedance indices were lower in AD patients, when comparing this study by Hagströmer et al. with Nicander and Ollmar (2004).^9,65^ The RIX values were higher and PIX values were lower. It was also found that the TEWL reading increased and stratum corneum moisture was lower in AD patients because the skin barrier function was impaired. The AD patients then applied a moisturiser (Proderm), two to three times a day, for a 3-week period. Assessments on the 10th and 21st day were carried out. Upon application of moisturisers, some of the impedance indices of AD skin became more comparable with normal skin, which may reflect an improvement in skin condition. This included an increase in PIX and decrease in RIX indices, though IMIX and MIX were unchanged.^9^

In all five studies, a decrease in MIX correlated with an impaired skin barrier function (including AD sufferers and application of topical irritants to the skin).^9,64–67^ Where mentioned, IMIX was also found to display a decreased value in compromised skin.^9,65,67^ The trend in PIX varied depending on the context of study; SLS treatment increased the value of PIX^66,67^ but NAA treatment decreased it.^66^ The opposing trends in PIX for each irritant suggest that the skin reactions can be distinguished. Although the study by Kuzmina et al. showed consistent PIX trends in two laboratories, more comparative studies on the reaction of skin to specific irritants such as SLS and NAA are needed.^66^ If it is possible to distinguish between the effects of different irritants in terms of skin impedance, this could lead to precise diagnosis of ailments concerning skin barrier function. This is not currently possible with standard techniques, such as TEWL. Assessment of AD skin compared to healthy skin also gave varied results; Nicander and Ollmar reported an increase in PIX on atopic skin whilst Hagströmer et al. reported the opposite trend.^9,65^ In terms of RIX, SLS treatment caused an increase, whilst tape stripping of both normal and AD skin caused a decrease.^26,28^ Furthermore, AD skin had a decreased value of RIX compared to healthy skin.^9^

Overall, the five impedance indices studies reflect a potential validity for skin impedance to monitor skin barrier function in dermatitis sufferers. There are some clear consistencies in impedance patterns with regards to skin integrity, particularly the MIX index. It has even been suggested by Nicander and Ollmar that impedance is the most sensitive technique which distinguishes between normal and atopic skin, when compared to standard techniques (electrical capacitance and TEWL).^26^ Kuzmina et al. noted that distinct changes in impedance indices may correspond to different irritants, detecting changes barely within range of visual detection. However, more research into the factors which affect skin impedance and the associated indices is still required.^66^ As well as the types of indices, it is unclear how skin properties vary at different depths; the evaluated studies measured impedance at a range of depth settings. It is also worth noting that these studies are limited to analysing the impedance parameters at two frequency points: 20 and 500 kHz. Use of the whole impedance spectrum would likely improve the utility of impedance measurements.

### Measuring impedance at frequencies of1 kHz–2.5 MHz

Rinaldi et al. carried out impedance spectroscopy measurements using Nevisense (Scibase), at 35 logarithmically-distributed frequencies between 1 kHz–2.5 MHz. Unlike the other studies, where impedance was only analysed at two frequency points, Rinaldi et al. used an artificial intelligence-based algorithms to generate an EIS score between 0 and 1 which encompassed the frequency range. The lower the EIS score, the weaker the skin barrier function. The aim of the work was to differentiate between AD patients (lesioned and non-lesioned skin) and healthy participants. This study involved the recruitment of 36 AD patients, during a 3-week hospitalisation period, and 28 healthy participants were recruited. EIS showed a significant inverse relationship with TEWL and was even found to be more sensitive than TEWL in terms of discriminating the healthy controls from non-lesioned AD.^68^ This observation bears similarities with the study by Nicander and Ollmar where it was also found that impedance was the most useful technique to identify differences between normal and atopic skin.^26^ During the 3-week period, Rinaldi et al. found that lesions showed an increase in EIS which indicates healing. Furthermore, EIS showed an inverse correlation with inflammatory biomarkers, including cytokines and chemokines.^68^ Overall, the study is very useful in providing the first example of the utility of impedance spectroscopy in monitoring dermatitis, where an extensive frequency range is used as opposed to just 20 and 500 kHz. It is vital that more research in this area is carried out to provide more confidence in the validity of this technique.

#### Using impedance spectroscopy to monitor IAD

Based on the available literature, impedance spectroscopy appears to be a good candidate for future investigations of IAD, from both a diagnostic and mechanistic perspective. In terms of diagnosis, impedance spectroscopy has proved to be successful in distinguishing patients with non-IAD dermatitis compared to normal healthy skin. These results are comparable or sometimes even superior to standard measurements of skin barrier function, such as TEWL.^9,64–68^ Therefore, it is likely that impedance spectroscopy could also be used to diagnose IAD, providing vital support to the current standard method (visual assessment). Mechanistically, there are several gaps in understanding of manifestation and progression of IAD. As discussed in Section 4, impedance spectroscopy could serve as a useful tool to dissect the contributions of each step in the IAD pathway (Figure 1) and develop specific therapeutic strategies.^16^

#### Limitations of impedance spectroscopy

As is the challenge with several skin barrier monitoring devices, including TEWL, impedance spectroscopy is currently unable to accurately differentiate between different damage mechanisms. Although Kuzmina et al. have suggested that impedance indices can be used to differentiate between dermatitis reactions caused by distinct irritants, the mechanistic details are not understood at present.^9,66^ It is likely that impedance spectroscopy could serve a role in monitoring skin health in conjunction with clinical observation, particularly in darker skin tones where erythema of early-stage IAD is not always clear. However, at present, it does not appear to be able to replace the need for visual assessment.

Furthermore, translation of the impedance spectroscopy technique into clinical practice may not be straightforward since products, such as protectants and barrier creams, applied to skin will have a significant effect on the measured skin impedance. It would therefore be necessary to assess the skin prior to reapplication of products. Also, the type of electrode systems used are important to consider. In all six studies reviewed in Section 3d on assessing dermatitis using impedance, the skin must be moistened with a physiological saline solution prior to applying the impedance probe. This step is necessary to improve skin to electrode contact in dry electrode systems but may be associated with variability, such as uneven pressure on the probe and skin wetting conditions.^66^ Furthermore, from a clinical perspective it would be disruptive and resource intensive to soak the skin in saline solution prior to measurements. Alternatively, the use of wet electrodes, such as the pre-gelled Ag/AgCl electrodes mentioned in Section 4a, do not require the use of saline solution. However, the gel alters the electrical properties of the skin over time, both due to the contents of the gel and accumulation of sweat, meaning that they are not suitable for long-term use. In recent years, development of soft electrodes, such as tattoo-based and skin-like electrodes, may become a preferred sensor in the future.^47^ The cost associated with the material and hardware for impedance spectroscopy measurements could also limit its translation into IAD monitoring.

However, impedance spectroscopy does provide an extra tool with which to probe skin integrity and is arguably the only methodology which gives a definitive measure of stratum corneum integrity (resistance). It is a valuable research tool, but results should be interpreted with care and in tandem with clinical observation and potentially TEWL, moisture loss and histology.

### Detecting and monitoring IAD with impedance spectroscopy: Exploratory work and preliminary findings

The authors’ own ongoing research into the potential utility of using impedance spectroscopy to monitor IAD has shown the technique to be highly sensitive in an *in vivo* model on healthy adult volunteers. The work has been approved by the University of Bath Ethics Committee (EP 22 064). Pilot data on one Caucasian male, aged 51, is detailed in Section 4b.

#### Methods and materials

**Figure 3.**
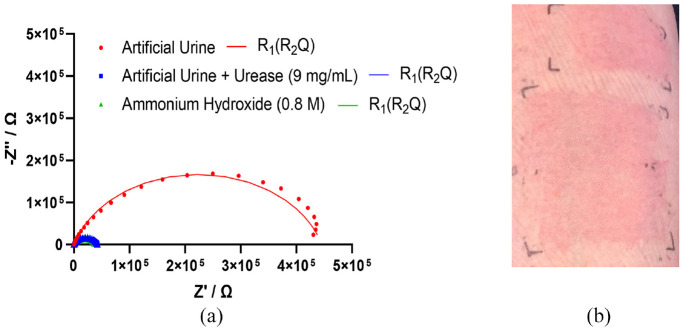
Nyquist plot, where Z′ is real part of impedance and −Z″ is the imaginary part of impedance, with fitting to an *R*_1_(*R*_2_*Q*) equivalent circuit model of skin exposed to artificial urine, artificial urine with urease from Canavalia ensiformis (9 mg/mL) or ammonium hydroxide (0.8 M) for 4 h (a) and visible erythema caused by urease (b).

Skin impedance was measured by placing two disposable Ag/AgCl pre-gelled ECG electrodes (3M, USA) directly adjacent to one another on the volunteers’ skin. Impedance was recorded between 50 kHz and 0.2 Hz, using the PalmSens4 potentiostat (PalmSens, The Netherlands). Using the PSTrace 5.8, data was fitted to a simple electrochemical equivalent circuit model, *R*_1_ (*R*_2_*Q*) circuit: *R*_1_ being the gel/cable resistance, *Q* the constant phase element relating primarily to the skin’s capacitance and *R*_2_ being attributed primarily to the stratum corneum resistance (Figure 3(a)). Ventral forearm skin was exposed to solutions of artificial urine,^69^ artificial urine with urease from *Canavalia ensiformis* (9 mg/mL) or ammonium hydroxide (0.8 M) for a total of 4 h.

#### Results and discussion

Exposure to artificial urine and purified urease from *C. ensiformis* created significant production of ammonia on the skin (detected by smell and rising skin pH), a decrease in skin impedance over the frequency spectrum (Figure 3(a)) and an erythematous rash (Figure 3(b)).

The stratum corneum is composed of dead keratinised cells which have an important barrier function and a high electrical resistance.^47,60,70^ Both the artificial urine with urease and the positive control (0.8 M ammonium hydroxide) caused a ten-fold reduction in stratum corneum resistance (*R*_2_) compared to the negative control (artificial urine only) (Table 1). This suggests that the presence of ammonia, produced by urease activity, damages the stratum corneum and causes the impedance pathway to shift to a lower resistance route.^60^

**Table 1.** The circuit element values of an *R*_1_(*R*_2_*Q*) equivalent circuit model of skin exposed to artificial urine, artificial urine with urease from *Canavalia ensiformis* (9 mg/mL) or ammonium hydroxide (0.8 M) for 4 h.

Condition	*R*_1_/Ω	*R*_2_/Ω	*Q*/µT	*n*/ϕ
Artificial Urine	1.54E + 02	7.63E + 05	0.08	0.85
	1.39E + 02	8.38E + 05	0.07	0.85
	1.54E + 02	4.46E + 05	0.10	0.82
Urease	1.58E + 02	4.81E + 04	0.19	0.79
	1.53E + 02	7.96E + 04	0.14	0.80
	1.35E + 02	4.22E + 04	0.18	0.79
Ammonium Hydroxide	8.73E + 01	3.41E + 04	0.23	0.74
	1.23E + 02	3.49E + 04	0.27	0.73
	1.35E + 02	3.95E + 04	0.27	0.75

The preliminary work by the authors shows that impedance spectroscopy could be a useful tool to monitor the progression of IAD and improve current understanding of its aetiology. A useful way to represent the multi-frequency impedance data is via equivalent circuit models. The study shows that urease activity plays an important role in the manifestation of IAD, due to the production of ammonia. The work is on-going and will be reported in full in 2023, including the use of urease-positive bacteria, addition of urease-inhibitors and faecal enzymes.

## Conclusions

There is clear evidence in the literature which demonstrates the utility of impedance spectroscopy in distinguishing between dermatitis/inflamed skin and normal healthy skin. The implementation of this technique in the detection of early-stage IAD could lead to a more accurate diagnosis and a significant reduction in both the prevalence and incidence of the cutaneous disorder. As previously mentioned, Kuzmina et al. noted that distinct changes in impedance may correspond to different irritants.^66^ This could be useful in distinguishing the effect of urinary and faecal irritants which are responsible for the development of IAD, compared to other causes of skin damage. Having tools which allow early diagnosis of IAD could reduce the financial strain on healthcare settings through earlier detection of impending skin disruption, allowing mitigating strategies to be deployed. Moreover, impedance spectroscopy gives the possibility of dissecting the relative role of the different agents involved in IAD which will allow a more detailed and complete picture of the pathogenic mechanism (Figure 1). Future work should involve exploring the role of urease activity in activating faecal enzymes, such as trypsin, α-chymotrypsin and lipase, and how urease inhibition could alleviate symptoms. Furthermore, the impedance detection system should be adapted for use in wearable equipment, allowing continuous measurement of skin health.^47,48^
